# Transcriptome Sequencing of the Spleen Reveals Antiviral Response Genes in Chickens Infected with CAstV

**DOI:** 10.3390/v13122374

**Published:** 2021-11-26

**Authors:** Joanna Sajewicz-Krukowska, Jan Paweł Jastrzębski, Maciej Grzybek, Katarzyna Domańska-Blicharz, Karolina Tarasiuk, Barbara Marzec-Kotarska

**Affiliations:** 1 Department of Poultry Diseases, National Veterinary Research Institute, 24-100 Puławy, Poland; domanska@piwet.pulawy.pl (K.D.-B.); karolina.tarasiuk@piwet.pulawy.pl (K.T.); 2 Department of Plant Physiology, Genetics and Biotechnology, Faculty of Biology and Biotechnology, University of Warmia and Mazury in Olsztyn, 10-719 Olsztyn, Poland; bioinformatyka@gmail.com; 3 Department of Tropical Parasitology, Institute of Maritime and Tropical Medicine, Medical University of Gdansk, 81-519 Gdynia, Poland; maciej.grzybek@gumed.edu.pl; 4 Department of Clinical Pathomorphology, The Medical University of Lublin, 20-090 Lublin, Poland; barbara.marzec@umlub.pl

**Keywords:** chicken astrovirus, white chicks syndrome, RNA-seq, molecular pathogenesis, differentially expressed genes, spleen transcriptome

## Abstract

Astrovirus infections pose a significant problem in the poultry industry, leading to multiple adverse effects such as a decreased egg production, breeding disorders, poor weight gain, and even increased mortality. The commonly observed chicken astrovirus (CAstV) was recently reported to be responsible for the “white chicks syndrome” associated with an increased embryo/chick mortality. CAstV-mediated pathogenesis in chickens occurs due to complex interactions between the infectious pathogen and the immune system. Many aspects of CAstV–chicken interactions remain unclear, and there is no information available regarding possible changes in gene expression in the chicken spleen in response to CAstV infection. We aim to investigate changes in gene expression triggered by CAstV infection. Ten 21-day-old SPF White Leghorn chickens were divided into two groups of five birds each. One group was inoculated with CAstV, and the other used as the negative control. At 4 days post infection, spleen samples were collected and immediately frozen at −70 °C for RNA isolation. We analyzed the isolated RNA, using RNA-seq to generate transcriptional profiles of the chickens’ spleens and identify differentially expressed genes (DEGs). The RNA-seq findings were verified by quantitative reverse-transcription PCR (qRT-PCR). A total of 31,959 genes was identified in response to CAstV infection. Eventually, 45 DEGs (*p*-value < 0.05; log_2_ fold change > 1) were recognized in the spleen after CAstV infection (26 upregulated DEGs and 19 downregulated DEGs). qRT-PCR performed on four genes (*IFIT5*, *OASL*, *RASD1*, and *DDX60*) confirmed the RNA-seq results. The most differentially expressed genes encode putative IFN-induced CAstV restriction factors. Most DEGs were associated with the RIG-I-like signaling pathway or more generally with an innate antiviral response (upregulated: *BLEC3*, *CMPK2*, *IFIT5*, *OASL*, *DDX60*, and *IFI6*; downregulated: *SPIK5*, *SELENOP*, *HSPA2*, *TMEM158*, *RASD1*, and *YWHAB*). The study provides a global analysis of host transcriptional changes that occur during CAstV infection in vivo and proves that, in the spleen, CAstV infection in chickens predominantly affects the cell cycle and immune signaling.

## 1. Introduction

Astroviruses cause enteritis in humans and other animals such as chickens, turkeys, sheep, cattle, swine, dogs, cats, and mice. In poultry, they cause enteritis combined with growth depression and a higher mortality, but their presence has also been described in healthy flocks [1]. The cause of such a phenomenon may be the existence of astroviruses of various virulence, but also the possibility of inducing a disease through the synergistic effect of several viruses simultaneously, e.g., astroviruses with rotaviruses or parvoviruses [2]. Furthermore, other enteric pathogens such as avian nephritis virus (ANV), avian orthoreoviruses, and fowl adenoviruses are often detected in co-infections with CAstV. CAstV infections spread mainly horizontally via the fecal–oral route. However, CAstV strains also transmit vertically from naive parent birds to chicks [3].

Astroviruses detected in various bird species belong to the *Astroviridae* family, genus *Avastrovirus*. Initially, they were classified into separate species depending on the host from which they were isolated. Since astroviruses may cross species barriers [4], the principles of their classification have been changed based on the amino acid structure of the viral capsid protein. Astroviruses detected in birds belong to three official species of astroviruses: one, two, and three [5].

Two different types of astroviruses, ANV and CAstV, have been identified in broiler chickens. ANV infection causes diarrhea, growth retardation, kidney damage, and gout, resulting in an increased mortality, especially in young chickens [6,7]. CAstV infections are associated with malabsorption syndrome/runting stunting syndrome [8,9,10,11]. CAstV can also cause pathology of the gastrointestinal tract (enteropathy), lameness, kidney inflammation, and embryo death. Astrovirus infections can significantly impact the poultry industry through a decreased egg production, breeding disorders, poor weight gain relative to feed intake, and increased mortality [12]. Relatively recently, CAstV has also been indicated as the causal factor of “white chicks syndrome” (WCS) affecting broiler chicks [13,14,15,16,17,18,19]. WCS is associated with an increased embryo/chick mortality, delayed hatching, weakness, and white plumage on hatched chicks. WCS can also induce subcutaneous edema and severe lesions in organs (mainly liver). To date, many aspects of CAstV–chicken interactions remain unclear, and there is no information available regarding gene expression changes in chicken spleen in response to CAstV infection.

The host’s ability to recognize and eliminate harmful pathogens is essential to its survival. These host–pathogen interactions are crucial in the disease course and are the subject of several studies that underline the essential role of the immune response and its antiviral receptors [20,21,22,23]. Virus–host interactions are determined mainly by pathogen virulence and host immune responses, which lead to changes in host gene expression [24]. Thus, pathogenesis involves complex interactions between the infectious pathogen and the immune system.

To activate an antiviral response, pattern recognition receptors (PRRs) must recognize specific pathogen-associated molecular patterns [25,26,27]. PRR activation leads to type I interferon (IFN) induction, cytokine secretion, and the activation of antigen-presenting cells. These, in turn, promote adaptive immune responses [28,29]. PRRs fall into four categories: the retinoic acid-inducible gene I (RIG-I)-like receptors, the Toll-like receptors, the NOD-like receptor, and the C-type lectin receptors. RIG-I-like receptors are ubiquitously expressed in the cytoplasm and comprise RIG-I, melanoma differentiation-associated gene 5 (MDA5), and the laboratory of genetics and physiology 2 (LGP2). RIG-I and MDA5 interact with the mitochondrial antiviral signaling gene *MAVS* [30], leading to transcriptional factor nuclear factor kappa B (NF-κB) activation and the production of type I IFNs and proinflammatory cytokines [31]. This condition then induces IFN-stimulated gene (ISG) expression to elicit antiviral effects. RIG-I and MDA5 are two key pattern recognition receptors that sense an RNA virus invasion, but RIG-I is absent in chickens. Although chickens have an intact *MDA5* gene, the genes acting downstream of chicken MDA5 that may mediate antiviral responses are not well studied [32].

The essential processes in living cells depend on the actions of proteins and ribonucleic acids (RNAs and DNAs). Knowledge about molecules involved in a particular function is essential to better understand and even modulate cell activity.

To better understand the interactions between the host and CAstV, we analyzed transcriptional profiles of the chickens’ spleens on the fourth day post infection (dpi) using RNA-seq. The sequencing results were compared and screened for differentially expressed genes (DEGs), using the Gene Ontology (GO) and Kyoto Encyclopedia of Genes and Genomes (KEGG) databases in the National Center for Biotechnology Information (NCBI). DEGs were verified by quantitative reverse-transcription PCR (qRT-PCR). Our results provide a foundation for future research on the pathogenesis of CAstV infection and may facilitate the discovery of candidate genes that can respond to and resist CAstV infection in chickens.

## 2. Materials and Methods

### 2.1. Virus and Animals

The CAstV strain PL/G059/2014 (GenBank accession no. JF414802), associated with “white chicks syndrome”, was propagated on embryonated specific pathogen-free (SPF) chicken eggs as described previously [16,19]. Internal organs of PL/G059/2014 chicken embryos homogenate were stored at −70 °C until further use.

SPF chicken embryos were obtained from VALO BioMedia (Osterholz-Scharmbeck, Germany) and housed in isolators until use.

### 2.2. Animal Experiments

Ten 21-day-old SPF White Leghorn chickens were divided into two groups of five birds each. One group was inoculated with CAstV, and the other was inoculated with sterile phosphate-buffered saline (PBS) and used as the negative control. Inoculations were given *per os* using 200 μL of a supernatant obtained after centrifugation of internal organs of PL/G059/2014 chicken embryos homogenized in PBS (20% *wt*/*vol*). Cloacal swabs were collected from infected chicks on the second and fourth days post inoculation to check for the presence of virus, as described in [33].

Chickens from each group were taken for necropsy at 4 dpi. Spleen samples were collected and immediately frozen at −70 °C for RNA isolation. Tissue samples were homogenized in RLT buffer containing β-mercaptoethanol, by use of an MP FastPrep-24 Tissue and Cell Homogenizer. Total RNA was extracted from 200 μL of tissue homogenate using a commercial kit (RNeasy Mini Kit; QIAGEN, Hilden, Germany) according to the manufacturer’s instructions.

### 2.3. RNA Quantification and Quality Control

RNA concentrations were measured using a Qubit RNA Assay Kit and a Qubit Fluorometer (Invitrogen, Carlsbad, CA, USA). RNA integrity was assessed using the RNA Nano 6000 Assay Kit of the Bioanalyzer 2100 system (Agilent Technologies, Santa Clara, CA, USA) for further cDNA synthesis and sequencing. The RNA integrity number (RIN) of each sample exceeded the threshold of 7.

### 2.4. Library Preparation for Transcriptome Sequencing

Ten cDNA libraries were created, using an external commercial service (Macrogen Europe B.V., Amsterdam, the Netherlands). The sequencing libraries were generated from total RNA, using a TruSeq Stranded mRNA LT Sample Prep Kit (Illumina, San Diego, CA, USA) according to the protocol in the TruSeq Stranded mRNA Sample Preparation Guide, Part #15031047 Rev. E. Libraries were sequenced on an Illumina NovaSeq 6000 System and 150 bp paired-end reads were generated.

### 2.5. Data Analysis

The quality control of both raw reads and trimmed reads was performed using FastQC v0.11.7 software [34]. The adapters and low-quality bases (average PHRED cut-off score < 30, cutting length up to 120 bp, minimal length 50 bp) were trimmed using Trimmomatic v0.38 [35]. The preprocessed paired-end reads were mapped to the reference chicken ENSEMBL genome (ver. GRCg6a.96) with the STAR (v2.7.0d) mapper [36]. The mapping results files were then merged using StringTie v1.3.5 [37], and expression in counts (number of reads aligned to the genome) and FPKM (normalized counts by the gene length and library depth) units were calculated using ballgown v2.14.1 [38].

The DEG analyses were performed using DESeq2 [39] in Bioconductor/R [40]. Only genes of adjusted *p*-value < 0.05 and absolute value of log_2_ fold change > 1 were classified as DEGs. The DEGs were subjected to functional enrichment analysis through the use of g:ProfileR [41] based on Gene Ontology (GO) [42] and the Kyoto Encyclopedia of Genes and Genomes (KEGG) [43] databases.

### 2.6. Accession Number

The raw sequencing data obtained in this study were submitted to the Sequence Read Archive (SRA) database under accession number PRJNA768620. The sequences were released on 30 October 2021.

### 2.7. qRT-PCR for Confirmation

Four immune-related genes (*OASL*, *IFIT5*, *RASD1*, *DDX60*) were selected for confirmation of the RNA-seq results. Five control and five infected chickens were subjected to the study. Each sample was analyzed in duplicate. The primers and probes used for qRT-PCR assays are listed in Table 1. qRT-PCR was performed in a reaction volume of 20 μL with the 7500 Real-Time PCR System (Applied Biosystems, Foster City, CA, USA), using the QuantiTect Probe RT-PCR Kit (QIAGEN) according to the manufacturer’s instructions. Primers and probes used in the study were previously described or are commercially available [44,45] (Table 1). The PCR cycling conditions were the following: one cycle of 50 °C for 30 min and one cycle of 95 °C for 15 min, followed by 95 °C for 15 s and 60 °C for 60 s for 40 cycles.

### 2.8. Statistical Analysis of qRT-PCR

The relative levels of expression of the target genes in the infected and control groups were calculated using the 2^−ΔΔCt^ method and quantified relative to β-actin. β-actin was used as a housekeeping gene to normalize the expression levels of the target genes, which were then expressed as the fold change in gene expression.

## 3. Results

### 3.1. Clinical Features of the CAstV-infected Chickens

Chickens infected with CAstV showed no symptoms for up to 4 dpi. We also did not observe any visible differences in the spleens between the control and infection groups. However, the CAstV did replicate in the infected chickens, as viral RNA was detected in cloacal swabs at 4 dpi.

### 3.2. Differential Expression Analysis

**Figure 1:**
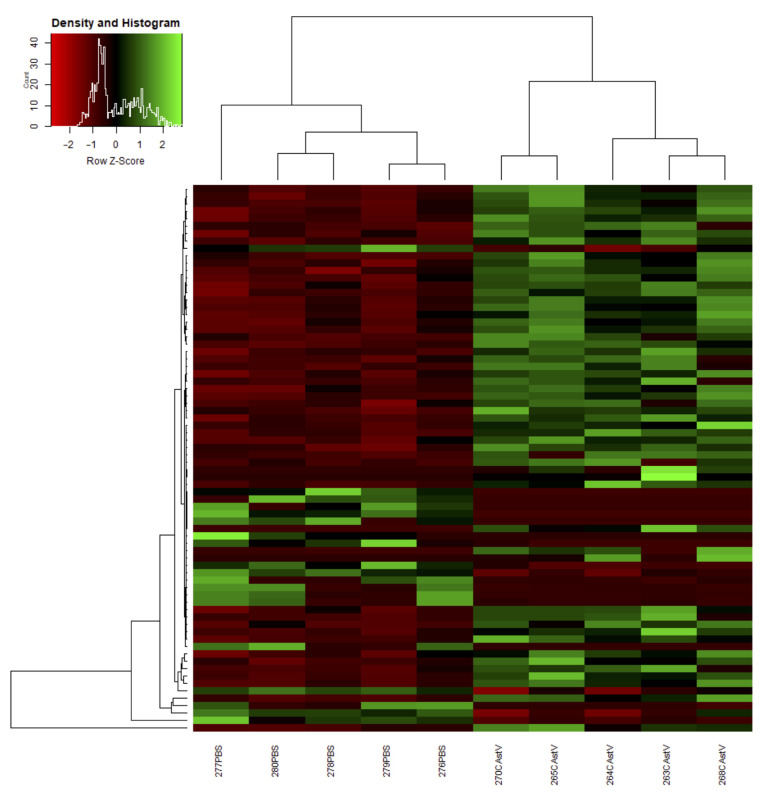
Heat map analysis used to classify gene expression patterns under different experimental conditions. Genes with similar expression patterns were clustered in the heat map. Intensity of color indicates gene expression levels. Red represents genes with high levels of expression and green represents genes with low levels of expression.

**Figure 2:**
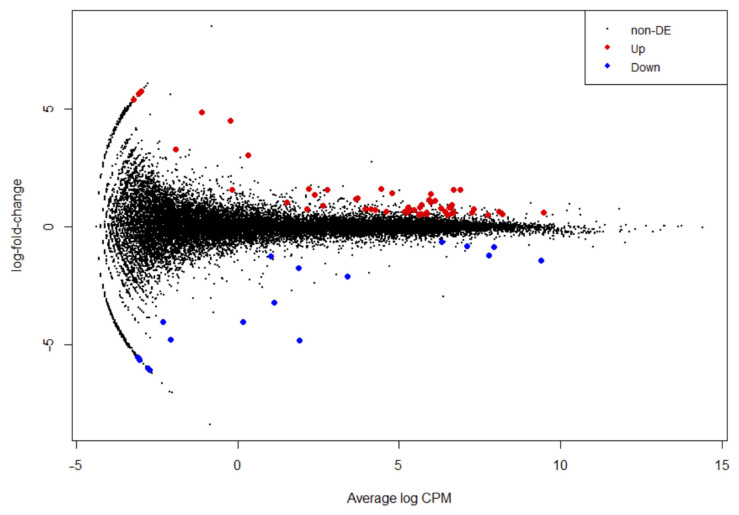
A volcano plot displays the number of DEGs between the control and CAstV-infected groups. Red points represent up-regulated genes, blue points represent down-regulated genes, and black points represent genes with no significant difference in expression.

A total of 393,831,314 reads was produced, comprising 58.9 Gbp. To ensure the best results for a further analysis, raw reads were filtered to remove low-quality data, with a total of over 338.4 million clean reads acquired, a mean of 90.2% of which was mapped to the chicken reference genome. The statistical metrics for the RNA libraries are listed in Table 2. We analyzed the DEGs with the DESeq R package. A total of 31,959 genes were identified in response to CAstV infection. When comparing the control and virus-infected groups, 204 significantly differentially (*p*-value < 0.05) expressed transcripts were noted, with 152 up-regulated (log_2_ fold change > 0) and 52 down-regulated (log_2_ fold change < 0). Subsequently, the transcripts were filtered using the thresholds of *p*-value < 0.05 and log_2_ fold change > 1. Under these criteria, 45 DEGs were identified in the spleen after CAstV infection (26 upregulated DEGs and 19 downregulated DEGs). These results were clearly visualized by clustering the samples by the differential treatment (Figure 1) and by constructing an MA plot of the DEGs (Figure 2). A list of the DEGs is provided in Supplementary File S1.

### 3.3. GO Analysis of DEGs after CAstV Infection

To identify differentially expressed gene functions, a gene ontology (GO) analysis was performed. This allowed us to categories and annotate DEGs into three groups: biological processes, molecular functions, and cellular components. Different gene functional distributions were noted upon comparing the transcriptional profiles of the CAstV-infected chickens with the control. The most enriched biological processes were those related to the cell cycle, the immune response, and the regulation of biological processes. Among the molecular functions group, the most enriched were processes, with the most significant molecular functions being the binding activity, including ATP binding. The cellular component of the GO analysis showed that the majority of enriched categories was relevant to intracellular components, such as chromosomes, kinetochores, or the microtubule cytoskeleton. A list of all GO terms has been listed in Supplementary File S2.

### 3.4. Pathway Enrichment after CAstV Infection in the Chicken Spleen

The KEGG database was used to analyze pathways to further define DEG functions in the chicken spleen after CAstV infection. At 4 dpi, there were significant changes in the mRNA levels of a set of genes in distinct pathway categories. We found that the virus elicited the enrichment of genes associated with immune-related pathways, including the NOD-like receptor signaling pathway and influenza A pathway, and the cell cycle. These three significantly enriched KEGG pathways are listed in Table 3 according to their *p*-values < 0.05. 

DEGs belonging to immune-related pathways and those associated with the cell cycle are listed in Table 4.

### 3.5. Verification of DEGs by qRT-PCR

In order to further confirm the differential gene expression obtained from the transcriptome sequencing data, we used qRT-PCR to analyze the expression levels of four selected genes (*OASL*, *IFIT5*, *RASD1*, *DDX60*). The gene selection was based not only on their log_2_ fold change and *p*-value threshold, but also on their biological relevance.

**Figure 3:**
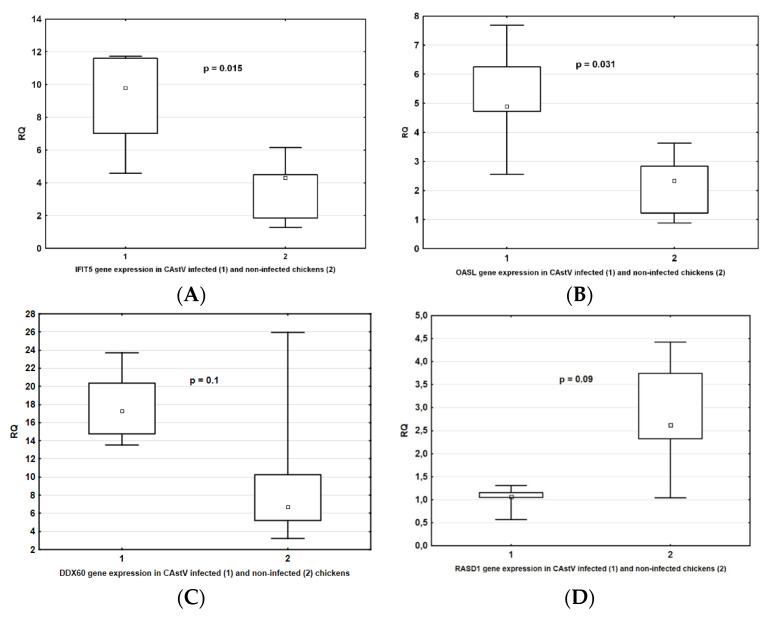
*IFIT* (**A**), *OASL* (**B**), *DDX60* (**C**), and *RASD1* (**D**) gene expression in CAstV-infected chickens in relation to non-infected chickens, as verified by qRT-PCR.

As shown in Figure 3 and Table 5, the expression of two genes (*IFIT*, *OASL*) differed significantly between the two studied groups, while two others (*RASD1* and *DDX60*) showed only a trend, possibly due to the small cohort sizes, toward a difference in both the RNA-seq and qRT-PCR analyses. These results supported the reliability of the differential expression identified using RNA-seq.

## 4. Discussion

White chicks syndrome (WCS) has recently been associated with CAstV. Ongoing outbreaks of WCS have been reported in Canada, Brazil, and some countries in Europe, including Poland [16,18,46,47]. Studies have described an increased embryo mortality in hatcheries. Hatched birds are sick and characterized by general weakness and white fluff. These birds rarely survive for more than 24 h. Molecular studies have found CAstV RNA in tissues of infected chickens [11].

Many reports have analyzed the transcriptomes of various avian tissues in viral infections [48,49,50,51,52,53], providing primary data for understanding the molecular mechanisms of viral infections in birds. Previous studies on CAstV focused mainly on the molecular characterization of the strains circulating in the field, while the biological processes, including immunological aspects and the cellular impact of WCS, or any CAstV infection, are poorly understood and studied.

Only a few studies on the immunology of TAstV (turkey astrovirus) infection in turkeys [54,55] and cytokine expression in day-old chicks with WCS have been published [46]. Here, to further facilitate investigations into CAstV pathogenesis and chickens’ response to infection, we performed a spleen transcriptome analysis in uninfected chickens and in chickens 4 dpi following infection with a WCS CAstV strain.

The global profile of gene expression in the spleen provided a good overview of the host response to CAstV infection. We chose the spleen because it is a secondary lymphoid organ that can effectively induce innate and adaptive immune responses that are especially important in birds because their lymphatic vessels and lymph nodes are underdeveloped [56].

Using RNA-seq, we identified DEGs in the chicken spleen during infection, focusing on the genes with a differential expression meeting the FC threshold (FC ≥ 1 or ≤ −1) for a further analysis. Numerous genes displayed significantly altered expression levels in the CAstV-infected chickens, including several that may be associated with intracellular responses to infection by chicken astrovirus (*BLEC3*, *CMPK2*, *IFIT5*, *OASL*, *DDX60*, *AVD*, *GZMA*, *LYGL*, and *IFI6*). Most of them are associated with the RIG-I-like signaling pathway or more generally with the innate antiviral response.

Interestingly, *BLEC3* is predicted to have immune functions. The exact functions of *BLEC3* in the anti-virus response are unknown, but it is probably an early activation antigen that may signal by binding *BLEC2* [57]. *CMPK2* is involved in HIV restriction in humans [58]. Our results may indicate that *CMPK2* upregulation inhibits the virus in infected chickens. The next overexpressed gene, *DDX60*, has been shown to be involved in both RIG-I activation and in RIG-I independent viral RNA degradation [59]. *IFIT5* is another antiviral response gene, with activity against RNA viruses such as orthomyxoviruses (e.g., avian influenza viruses) and paramyxoviruses (e.g., Newcastle disease virus) in chickens and Tembusu virus in ducks [60,61]. *OASL* is an important and possibly RIG-I-dependent inhibitor of RNA virus replication that prevents the virus from escaping from innate immunity. Ren et al. have reported that the newly described Chinese goose astrovirus, GAstV-GD, can induce a high-level expression of *OASL*, an essential host factor that limits viral replication in LMH cells (a chicken liver cell line) [62,63]. Our results suggest that CAstV is also effective in increasing *OASL* mRNA levels in vivo. The *AVD* gene encodes avidin, a likely host defense factor in chickens [64,65]. It inhibits microbial growth by binding to the biotin required by most bacteria as an essential enzyme cofactor. Lysozyme hydrolyses glycosidic bonds in bacterial peptidoglycan and is used as a natural preservative in meat products [66]. The antiviral spectrum of lysozyme is much less well known and mainly includes HSV1 and HIV-1 [67]. The strongly increased expression of the *AVD* and *LYGL* (Lysozyme G-like) genes after CAstV infection may be additional evidence of their antiviral function. Granzyme A is secreted by cytotoxic lymphocytes and mediates cell death and promotes inflammation. It also plays an important role in antiviral immunity, being necessary for the control of viral replication [68], and is significantly increased in the sera of patients and in the NK cells of mice infected with CHIKV [69]. Finally, *IFI6* delays apoptosis [70], lowers the titer of yellow fever virus in cell cultures, and strongly regulates Dengue 2 virus and West Nile virus infections [71,72].

Here, we provided in vivo evidence suggesting that these most-overexpressed DEGs were putative IFN-induced CAstV restriction factors. A better understanding of viral restriction may be helpful in developing new targeted therapies capable of controlling CAstV and other viral infections. Future studies should focus on understanding the degree of adaptive pressure these genes exert on CAstV. Their resistance to antiviral agents is probably the best measure of their importance [58].

In our study, we found that CAstV also significantly suppressed the mRNA levels of several genes (*SPIK5*, *SELENOP2*, *HSPA2* (*HSP70*), *TMEM158*, *RASD1*, and *14-3-3β* (*YWHAB*)).

In humans, the *SPIK5* gene encodes proteins involved in inhibiting the immune and inflammatory responses in human primary keratinocytes and mucous epithelium [73]. In chickens, the product of the *SPIK5* gene, ovoinhibitor, plays a significant role in the antibacterial defense of eggs against *Bacillus* spp. Moreover, the downregulation of *SPIK5* expression after ILTV vaccination induces immune responses [74,75]. We also found a significant downregulation of the expression of the selenoprotein P (*SELENOP*) gene, which is involved in the antioxidant, anti-inflammatory, and antiviral activities of selenium. RNA viruses, including coxsackievirus B3 and influenza A H3N2, can mutate into more virulent strains in a selenium-deficient host [76,77]. It has also been shown that severe serum selenium and *SELENOP* deficiency in patients with COVID-19 is associated with a higher risk of dying from COVID-19 [78]. Murai et al. shown that the expression of selenoprotein P mRNA in the liver increases after HCV infection [79]. *SELENOP* mRNA suppresses the response of type I interferon by inhibiting the properties of RIG-I. Conversely, the downregulation of *SELENOP* mRNA in our study probably caused a strong induction of the RIG-I-like pathway and, consequently, interferon-stimulated genes [79]. Heat-shock proteins (HSPs), including HSP70, are essential for cell survival during stress. Infection by viruses induces HSP expression and facilitates viral production [80]. HSP70 is involved in viral entry to the cell, uncoating, and genome replication and expression [81,82]. It is associated with the formation of the viral replication–transcription complex and regulates the replication of hepatitis C virus [83], flock house virus [84], and herpes simplex virus type 1 [85]. We also found that the mRNA level of *RASD1*, which encodes a member of the RAS superfamily of small G-proteins, was decreased. The role of the *RASD1* protein in host–virus interactions is not entirely clear. However, there is evidence to suggest that the activation of the Ras pathway is essential for an efficient viral protein synthesis [86]. We did not find examples in the literature describing the action of *TMEM158* in viral infection. However, Cheng et al. showed that *TMEM158* expression notably inhibits the proliferation, cell cycle progression, adhesion, invasion, and tumorigenicity of ovarian cells [87]. The silencing of *TMEM158* in ovarian cancer cells promoted a G1-phase arrest, which may inhibit cell proliferation. Based on the results of our research, it can be assumed that the downregulation of *TMEM158* gene expression after astrovirus infection may also lead to the negative regulation of the cell cycle and cell-cycle arrest [88]. The 14-3-3 protein family plays a key role in the regulation of intracellular signaling pathways [89]. They also participate in the control of the cell cycle, metabolism, apoptosis, and gene transcription [90]. They influence a variety of signal transduction pathways and are targeted by viruses that modulate their activity to alter cellular processes and facilitate viral invasion. The 14-3-3 protein interacts with the HIV Vpr protein and regulates the cell cycle by binding to Cdc25C and inducing a G2-M arrest during HIV infection [91]. Moreover, the NS3 protein of dengue, Zika, and West Nile viruses binds to 14-3-3ε/η and prevents RIG-I translocation to the adaptor protein, thereby blocking antiviral signaling in humans [92,93,94]. Little is known about the role of the 14-3-3β protein in chickens, but we can assume that it is analogous to that of other isoforms.

The next step in our study was the enrichment analysis of DEGs. We analyzed the pathway enrichment, the biological functions and the molecular processes in which these pathways are involved. Our goal was to understand the host responded to CAstV infection, so pathway analysis alone might not have been sufficient. The NOD-1 signaling pathway involved extremely dynamic molecular interactions. Detecting alterations in this pathway did not necessarily provide a complete picture of the biological effects. Moreover, the KEGG analysis revealed three enriched pathways, compared with several hundred biological processes.

The GO analysis showed that the most upregulated genes were mainly involved in immunity and defense, and the response to and regulation of viral/biological processes, while the downregulated gene functions were mainly related to the regulation of biological processes. Some were not classified into any biological process.

KEGG signaling pathway annotations to the DEGs showed that the expression of immune response-related genes at 4 dpi mainly involved the NOD-like receptor signaling pathway and influenza A pathway. A third group of DEGs was also enriched in the cell cycle pathway.

We used qRT-PCR to verify the relative expression of four immunity-related genes. The results showed that the expression of these genes was consistent with the transcriptome sequencing results.

Interactions between the host and the virus included cellular and immune responses and the countermeasures used by the viruses themselves. A small viral genome and high replication and mutation rates present a constant challenge to the host. Thus, viruses either evade detection or modulate host physiology to make cellular pathways work to their advantage. To avoid detection, they can impair host cellular processes such as cell cycle regulation, including checkpoint deregulation [95], major histocompatibility complex-restricted antigen presentation, intracellular protein transport, apoptosis, cytokine-mediated signaling, and humoral responses.

The interaction of a virus with the cell cycle can have various effects, such as promoting viral DNA genome replication or a cell cycle delay to allow sufficient time for RNA virus assembly [95]. Cell cycle control is well characterized in DNA viruses and retroviruses, whose primary replication site is the nucleus. Little is known in the case of RNA viruses, and especially the poorly characterized chicken astroviruses, whose primary replication site is the cytoplasm [96]. The RNA virus alteration of the host cell cycle and its mechanisms are not well characterized. It is assumed that a cell cycle arrest can benefit viral replication. For the negative-strand RNA viruses, there are several examples of cell cycle control. For instance, measles virus infection results in a G0 block, and the paramyxovirus simian virus V protein prolongs the cell cycle by delaying cells in G1 and G2 [97,98]. In the case of positive-strand RNA viruses, the avian coronavirus infectious bronchitis virus delays cell growth by inhibiting cytokinesis and also allows cells to accumulate in S/G2 [98]. The avian coronavirus infectious bronchitis virus also promotes favorable conditions for viral protein synthesis and, hence, progeny virus production, by inducing a cell cycle G2/M phase arrest of virus-infected cells [99].

We found that CAstV infection promoted a cell cycle arrest in spleen cells. This cell cycle arrest was accompanied by the inhibition of genes encoding TMEM158 and YWHAB (belonging to the 14-3-3 protein family) and a significant increase in cyclin-dependent kinase inhibitor 2C (*CDKN2C*) mRNA [100]. *CDKN2C* encodes an inhibitor that prevents the activation of the cyclin-dependent kinases (CDKs) and controls the G1 phase of the cell cycle. CDKs are key regulators of transitions from one phase of the cell cycle to the next [101]. However, further investigations are required to clarify the relevance of this regulation to CAstV replication.

The enrichment analyses demonstrated that CAstV infection caused the increased expression of mainly ISGs involved in various signaling pathways, primarily the RIG-I-like signaling pathway. The mRNA levels of cell cycle and stress response genes were mostly decreased. Our results demonstrated that CAstV infection triggered innate responses in the chicken spleen and revealed a series of ISGs that interact during viral infection. We hypothesized that CAstV has a mechanism of immune evasion that prevents the significant activation of immune genes in the early stages of infection, but this may also depend on the virulence of the virus and the age of the chickens. Studies on turkey astrovirus show that innate immune responses are crucial in fighting against astrovirus infections in young birds [63,102]. Turkey astrovirus type 2 (TAstV-2), isolated from turkeys diagnosed with poult enteritis and mortality syndrome and similar to human astrovirus, exhibits immunomodulatory properties. Qureshi et al. demonstrated that TAstV-2-infected chicken lymphocytes have a significantly decreased mitogen response and suggested that this could lead to severe immune disorders [103]. In subsequent studies, they proved that TAstV-2 infection causes changes in innate immune cells. These cells were characterized by decreased phagocytic activity, decreased bacterial killing activity, and decreased expression of the proinflammatory cytokines IL-1 and IL-6 [104], suggesting that TAstV-2 infection suppresses the innate immune system.

Interestingly, our KEGG analysis revealed that *JUN* gene downregulation was common to both of the enriched pathways associated with the antiviral response (the NOD-like receptor signaling pathway and influenza A pathway). c-jun is a downstream component of the c-jun N-terminal Kinases (JNKs) signaling pathway and a crucial cofactor of activator protein AP-1. It may take part in the development of viral infections [105] and can be activated by many extracellular factors, including viral infections [106]. For example, in influenza A virus (IAV) infection, the *JUN* gene is phosphorylated and, thus, activated at a very early stage [107]. This enables the further initiation of antiviral agents, including IFNβ, which in turn can cause significant inflammation [108]. Xie et al. demonstrated that suppressing *JUN* in human and mouse cells efficiently reduces IAV replication and resets the balance of pro- and anti-inflammation induced by IAV infection, both in vitro and in vivo [105]. Thus, the suppression of *JUN* gene expression in CastV infection may also be evidence of the immunosuppressive effect of CAstV.

Another example of the immunosuppressive nature of CAstV infection appears to be its effect on the complement system, which helps the avian immune system fight viral infections. As part of the innate immune response, it is immediately ready to target and eliminate virus particles and interact with virus-infected cell surfaces [109]. Growing evidence suggests that RNA viruses, such as their DNA counterparts, have evolved strategies to restrict complement function by modulating the expression of host genes involved in the antiviral response [110]. Infection with avian astroviruses, such as human ones, is accompanied by limited inflammation. This suggests that astroviruses may somehow disrupt the innate immune system during infection [102,111]. Indeed, both HAstV and its purified recombinant CP protein have been shown to inhibit serum complement activation [112,113]. HAstV CP inhibits the activation of the classical and lectin complement pathways. In the classical pathway, the CP protein inhibits C1 activation by directly binding to C1q and displacing the C1s–C1r–C1r–C1s serine protease tetramer [113]. In the lectin pathway, CP inhibits the activation of mannose-binding lectin by binding directly to it at the same binding site as the serine protease MASP-2 [114,115]). Our analysis revealed that the *C1R* and *C1S* genes of the complement pathway were upregulated at the 4 dpi time point (log_2_ fold change = 0.8).

The lack of the activation of other components of the complement system suggests a possible inhibition of the pathway at this stage by CAstV. The crucial role of complement is to initiate an inflammatory response. The absence of inflammation in astrovirus infection suggests that the suppression of complement activation by astroviruses is an essential component of immune and inflammatory response suppression. Indeed, research does seem to confirm this. Tam et al. found only low levels of the complement-mediated activation of NF-κB after HAstV infection in comparison with adenovirus and human papillomavirus infection, suggesting that HAstV has complement avoidance strategies [116,117]. Moreover, the lack of C3 activation, which is needed to trigger an interferon response, may explain the lack of activation of IFNs and, consequently, cytokines in our experiment. A similar situation has been described by Guix et al. and by Marvin et al. in studies using the human astrovirus [117,118].

## 5. Conclusions

In conclusion, this study provided broad insight into the CAstV-induced host response and a basis for further studies that may clarify the interactions between virus and host. Although this study had some limitations, such as using small study groups and a single time point, it, nonetheless, provided a global analysis of host transcriptional changes that occur during CAstV infection in vivo, new information about novel genes in chickens, and a strong basis for further studies.

We demonstrated that CAstV infection in chickens affects both the cell cycle and immune signaling in the spleen. We believe that the results regarding molecular mechanisms and the host immune response warrant a further transcriptomic analysis at multiple time points after infection.

## Figures and Tables

**Table 1:** Primers and probes used in the study.

Target Gene	Sequence (5′–3′)	References
*OASL*	F: AGCACTGGTACAAGGAGATGTTG	Cong et al., 2013 [44]
	R: CCAAGCAGCTCCAGCACAG	
	P: CTGAAGTCCTCCCTGCCTGTGCCCT	
*IFIT5*	F: AAAAGAAGGCAAATCATGAGTACC	
	R: TGATCCTCTATTGATTCTTCCAGAC	
	P: AATTCCTTGAAGAACTCCCTGCTGC	
*ACTB*	F: CATCCTCACCCTGAAGTACC	Vora et al., 2004 [45]
	R: GCTCATTGTAGAAGGTGTGG	
	P: CACGGCATCGTCACCAACTG	
*DDX60*	N/A	Thermo Fisher, Gg07198553_m1
*RASD1*	N/A	Thermo Fisher, Gg03359818_g1
*YWHAB*	N/A	Thermo Fisher, Gg03369026_m1
*HSPA2*	N/A	Thermo Fisher, Gg03370143_s1

**Table 2:** The statistical metrics for the RNA libraries. PBS refers to control samples; CAstV refers to experimental samples.

RNA-seq Libraries	Number of Raw Reads (Millions)	Number of Processed Reads (Millions)	Number of Uniquely Mapped Reads (Millions)	Uniquely Mapped Reads (%)
263CAstV4dpi	31.6	27.2	24.8	91.4
264CAstV4dpi	40.4	35.0	30.8	88.3
265CAstV4dpi	41.0	35.0	30.4	86.7
268CAstV4dpi	39.4	33.6	30.8	91.8
270CAstV4dpi	41.2	35.2	30.8	87.5
276PBS4dpi	36.2	30.8	28.0	90.7
277PBS4dpi	46.6	38.6	35.6	91.8
278PBS4dpi	35.4	31.8	29.2	91.7
279PBS4dpi	41.4	35.2	32.0	90.9
280PBS4dpi	40.6	36.0	32.8	91.4

**Table 3:** KEGG pathways enrichment in chickens infected with CAstV at 4 dpi.

Description	*p*-Value	Negative log_10_ of Adjusted *p*-Value
NOD-like receptor signaling pathway	0.023364421	1.631444986
Influenza A	0.025663115	1.590690622
Cell cycle	0.029423013	1.531312861

**Table 4:** DEGs associated with the immune pathway and cell cycle in the spleen transcriptomes of chicks infected with CAstV at 4 dpi.

NOD-like receptor signaling pathway	ENSGALG00000010870,ENSGALG00000017186,ENSGALG00000000720, ENSGALG00000007651,ENSGALG00000001619
Influenza A	ENSGALG00000010870,ENSGALG00000003584,ENSGALG00000007651, ENSGALG00000041192,ENSGALG00000003144
Cell cycle	ENSGALG00000004143,ENSGALG00000005769,ENSGALG00000008233, ENSGALG00000036892,ENSGALG00000010537

**Table 5:** IFIT, OASL, DDX60, and RASD1 expression in CAstV-infected and non-infected chickens, as verified by qRT-PCR.

Genes	CAstV Infected Chickens		CAstV Non-Infected Chickens		*p*-Value
	RQ Mean ± SD	RQ Median	RQ Mean ± SD	RQ Median	
*IFIT5*	8.9 ± 3.6	9.8	3.6 ± 2.0	4.3	0.015
*OASL*	5.2 ± 1.9	4.9	2.2 ± 1.1	2.3	0.015
*DDX60*	17.9 ± 4.2	17.3	10.3 ± 9.1	6.72	0.01
*RASD1*	1.03 ± 0.3	1.06	2.8 ± 1.3	2.62	0.09

## Data Availability

The raw data generated in RNA-seq study were submitted to the SRA database (https://www.ncbi.nlm.nih.gov/sra) (accessed on 30 October 2021) under accession number PRJNA768620 (release date: 30 October 2021).

## Supplementary Materials

The following are available online at www.mdpi.com/article/10.3390/v13122374/s1, Supplementary File S1: differentially expressed genes in CastV-infected group compared with control group, Supplementary File S2: CAstV-infected vs. control GO-enriched biological processes.
